# Transcriptome analyses identify key genes and potential mechanisms in a rat model of osteoarthritis

**DOI:** 10.1186/s13018-018-1019-3

**Published:** 2018-12-14

**Authors:** Hui-Zi Li, Hua-Ding Lu

**Affiliations:** 1 Department of Orthopaedics, The Fifth Affiliated Hospital of Sun Yat-Sen University, Zhuhai, 519000 Guangdong China; 2 Guangdong Provincial Engineering Research Center of Molecular Imaging, The Fifth Affiliated Hospital of Sun Yat-Sen University, Zhuhai, 519000 Guangdong Province China

**Keywords:** Osteoarthritis, Differentially expressed genes, Animal model, Bioinformatics analysis

## Abstract

**Background:**

Osteoarthritis (OA) is one of the most common degenerative diseases of the joints worldwide, but still the pathogenesis of OA is largely unknown. The purpose of our study is to clarify key candidate genes and relevant signaling pathways in a surgical-induced OA rat model.

**Methods:**

The microarray raw data of GSE8077 was downloaded from GEO datasets. GeoDiver were employed to screen differentially-expressed genes (DEGs). Enrichment analyses of DEGs were performed using Metascape. Construction of protein–protein interaction (PPI) network and identification of key genes were conducted using STRING, Cytoscape v3.6.0, and Centiscape2.2. Furthermore, miRDB and Cytoscape v3.6.0 were used for visualization of miRNA-mRNA regulatory network. Kyoto Encyclopedia of Genes and Genomes (KEGG) pathway analysis for predicted miRNAs was undertaken using DIANA-miRPath v3.0.

**Results:**

Several DEGs (188 in comparison between OA and sham-operated group and 160 in comparison between OA and contralateral group) were identified. DEGs mainly enriched in vasculature development, regulation of cell migration, response to growth factor (Gene ontology), and ECM-receptor interaction (KEGG). Two comparison cohorts shared 79 intersection genes, and of these, Ccl2, Col4a1, Col1a1, Aldh1a3, and Itga8 were defined as the hub genes. Predicted miRNAs of seven DEGs from sub-networks mainly enriched in MAPK signaling pathway.

**Conclusion:**

The current study shows that some key genes and pathways, such as Ccl2, Col4a1, Col1a1, Aldh1a3, Itga8, ECM-receptor interaction, and MAPK signaling pathway may be associated with OA progression and act as potential biomarkers and therapeutic targets for OA.

**Electronic supplementary material:**

The online version of this article (10.1186/s13018-018-1019-3) contains supplementary material, which is available to authorized users.

## Introduction

Osteoarthritis (OA), one of the most important causes leading to joint disability, is associated with increased social and medical burden [1, 2]. Regardless of wonderful advancements in diagnosis and treatment of OA these years, the prevalence of OA still increases from 6.6% to 14.3% between 1999 and 2014 in the USA [3, 4]. Currently, the main management for early-stage OA includes lifestyle modification and pharmaceutical drugs, such as regular physical activity, Tai Chi, non-steroidal anti-inflammatory drugs, intra-articular hyaluronic acid, and corticosteroid injections [5–9]. Irrespective of their potential effectiveness in increasing the time from diagnosis of OA to joint arthroplasty, these non-operative treatments can hardly block or reverse OA progression [10]. Eventually, total joint replacement was recommended by orthopedic surgeons for patients with advanced OA owing to serious radiographic grade, pain, and functional impairment of involved joints [11]. A crucial reason for these lies in that the key candidate genes and relevant signaling pathways associated with OA remains largely unknown. As a result, it is critical to further elucidate the pathogenesis of OA onset and progression.

Accumulative evidence suggested that many differentially expressed genes (DEGs) may participate in OA development. Kuttapitiya and colleagues demonstrated that 218 DEGs in bone marrow lesions of OA patients were related to OA-induced pain [12]. Ramos et al. suggested that 694 DEGs were identified in blood of OA patients and these DEGs mainly enriched in the apoptosis pathways, which may be associated with the onset of OA [13]. Recently, bioinformatics analysis were widely used to identify DEGs and perform subsequent enrichment analyses, such as Gene ontology (GO) and Kyoto Encyclopedia of Genes and Genomes (KEGG) pathway analysis, which may largely promote the understanding of OA pathogenesis [14, 15]. Surgical-induced OA rat model which mainly involves anterior cruciate ligament transaction and destabilizing medial meniscus, is frequently used to explore the pathogenesis of OA in vivo. Previous studies also indicated that several ectopically expressed genes, such as AQP-1, GDF5, and TAK1, participated in the development of surgically induced OA in rat models [16–18]. However, most of researchers merely attached importance to individual OA-related gene, which can hardly have a comprehensive understanding of corresponding molecule mechanisms, which were usually complicated and networked. Understandably, bioinformatics analysis may be a powerful way to explore these complicated regulatory networks and molecule mechanisms in a surgical-induced OA rat model.

In the current study, we identified several DEGs in a surgical-induced OA rat model after re-analyzing the raw microarray data (GEO Series: GSE8077). Enrichment analyses of DEGs were performed using Metascape. Construction of protein–protein interaction (PPI) network and identification of key genes were conducted using STRING, Cytoscape, and Centiscape2.2. Furthermore, miRDB and Cytoscape v3.6.0 was used for visualization of miRNA-mRNA regulatory network. We also performed KEGG pathway analysis for predicted miRNAs based on DIANA-miRPath v3.0.

## Materials and methods

### Microarray data

The microarray data (GSE 8077 or GDS2809) deposited by Appleton et al. was downloaded from GEO database (https://www.ncbi.nlm.nih.gov/geo/), which included three groups (surgical-induced OA group, contralateral group, and sham group) [19]. The expression data was generated on Affymetrix Rat Genome 230 2.0 Array platform [Rat230_2]. In the dataset, five rat models of OA were established through anterior cruciate ligament transection and partial medial meniscectomy. Another five rats were performed with sham surgery to act as control groups. After 4 weeks of forced mobilization, three times per week, cartilage of three groups was harvested for further experiments. Surgical-induced OA rat models were successfully induced at 4 weeks after operation, which were verified by Safranin O staining [19].

### Identification of differentially expressed genes

DEGs between OA and control group (Contralateral group or Sham group) were identified using GeoDiver (https://www.geodiver.co.uk/). Briefly, two comparison groups(OA vs. Contralateral group or Sham group) were assigned to analyze after loading the data of GDS2809 in GeoDiver. And then, DEGs were identified after clicking “ANALYSE GEO DATASET” with default parameters. DEGs with the cut-off criterion (P value < 0.05 and Log|FC|>1) were considered for further analyses. 

### Enrichment analysis for DEGs

Enrichment analyses for DEGs were performed using Metascape (http://metascape.org/gp/index.html#/main/step1), a powerful web-based tool, which involved in four processes: ID Conversion, Gene Annotation, Membership Analysis, and Enrichment Analysis [20]. The available terms for enrichment analysis includes pathway (Reactome Gene Sets, Canonical Pathways, BioCarta Gene Sets, GO Biological Processes, Hallmark Gene Sets, and KEGG Pathway), functional set (Go Molecular Functions), structural complex (Go Cellular Components, KEGG Structural Complex, and CORUM Protein Complex), and signature module (immunologic signatures, oncogenic signatures, and chemical and genetic perturbations). More interestingly, Metascape provides more frequently updated bioinformatics analyses than DAVID [21]. Three steps were followed to perform enrichment analyses for DEGs in the current study. Firstly, we undertook enrichment analysis for DEGs in two comparison cohorts independently. And then, enrichment analysis for the intersection genes across two comparison cohorts was performed. Finally, we conducted meta-enrichment analysis for two DEGs lists.

### Integration of protein–protein interaction (PPI) network and module analysis

PPI networks of intersection DEGs were analyzed with the threshold (combined_score > 0.4) using the STRING tool (http://www.string-db.org), which can provide interactions across matched proteins [22]. And then, Cytoscape v3.6.0 was employed to construct PPI networks [23]. Cytoscape plug-in CentiScaPe was used to assess the centrality of DEGs in PPI networks with three algorithms: degree centrality, betweenness centrality, and closeness centrality [24]. DEGs with centrality degree ≥ 3 were defined as key genes in the current study. Furthermore, the expression level of hub genes between sham group and OA group were compared with unpaired Student’s *t* test after extracting the original expression value in GEO Profiles (https://www.ncbi.nlm.nih.gov/geoprofiles/). *P* < 0.05 was thought to be statistically significant. All the statistical analyses were conducted using GraphPad Prism 7. Subsequently, molecular complex detection (MCODE) was applied to extract sub-networks in PPI networks with default algorithms (degree cut-off of 2, node score cut-off of 0.2, K-Core of 2, and max. depth of 100) [25].

### Construction of miRNA-mRNA regulatory network and identification of miRNA-associated pathways

MiRNAs, a class of non-coding RNA with 20–22 nucleotides (nts), can bind to the 3′UTR regions of targeted mRNAs to induce translational repression or degradation of mRNAs [26]. In the current study, miRNAs interacting with seven mRNAs from sub-networks (Itga8, Col1a1, Col12a1, Col4a1, Ccl7, Mmp12, and Ccl2) were predicted using an online database (miRDB) [27]. Cytoscape was used for construction of miRNA-mRNA regulatory network. We also performed KEGG analysis for predicted miRNAs based on DIANA-miRPath v3.0, a useful web tool which can provide experimentally supported miRNAs-mRNA interaction [28]. The results of KEGG enrichment for predicted miRNAs were visualized using package ggplot2 in R (https://www.r-project.org/).

## Results

### Analysis of DEGs

**Fig. 1:**
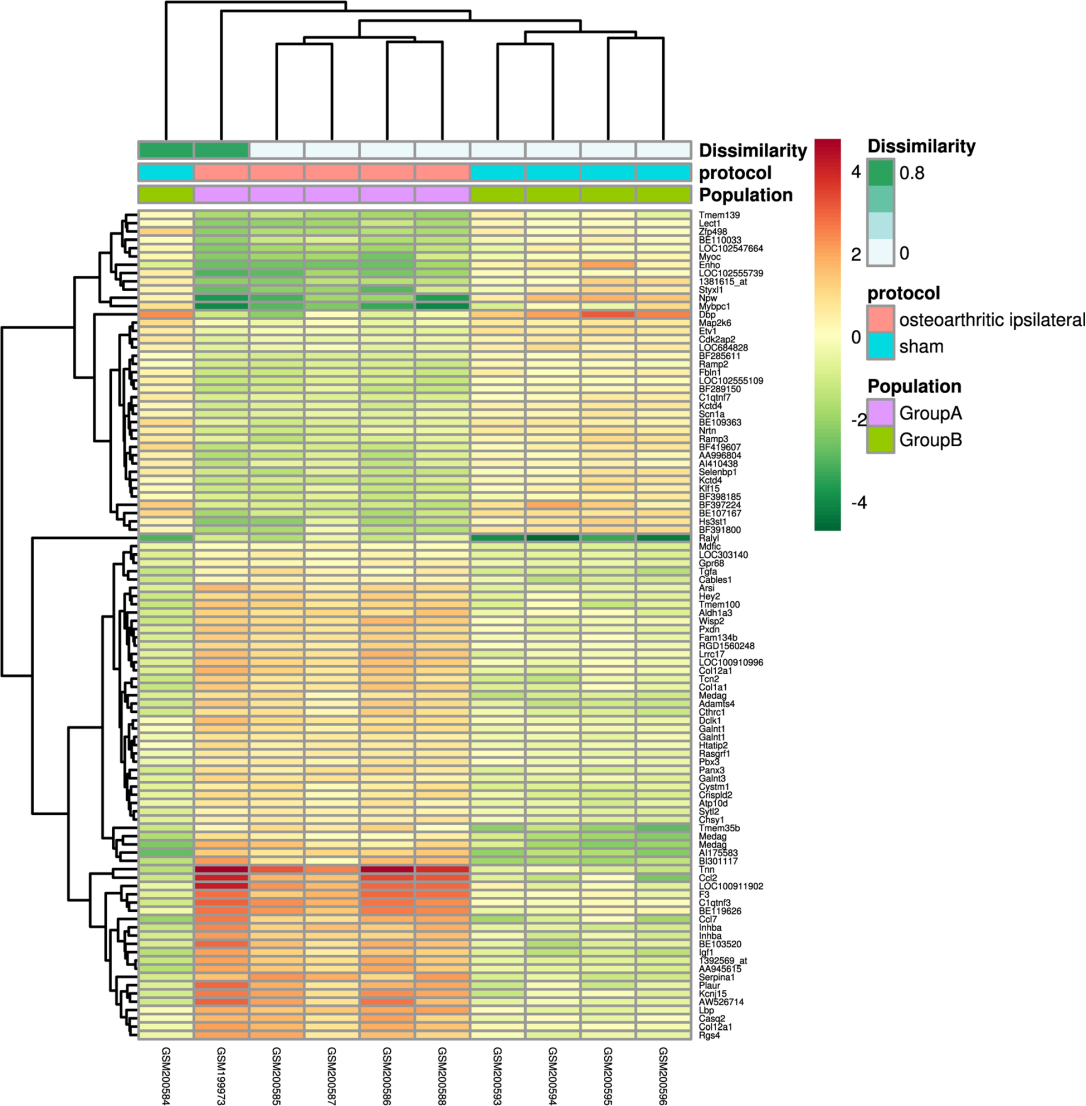
Heatmap of DEGs in comparison between OA group and sham group. The heatmap was produced using GeoDiver(https://www.geodiver.co.uk/)

**Fig. 2:**
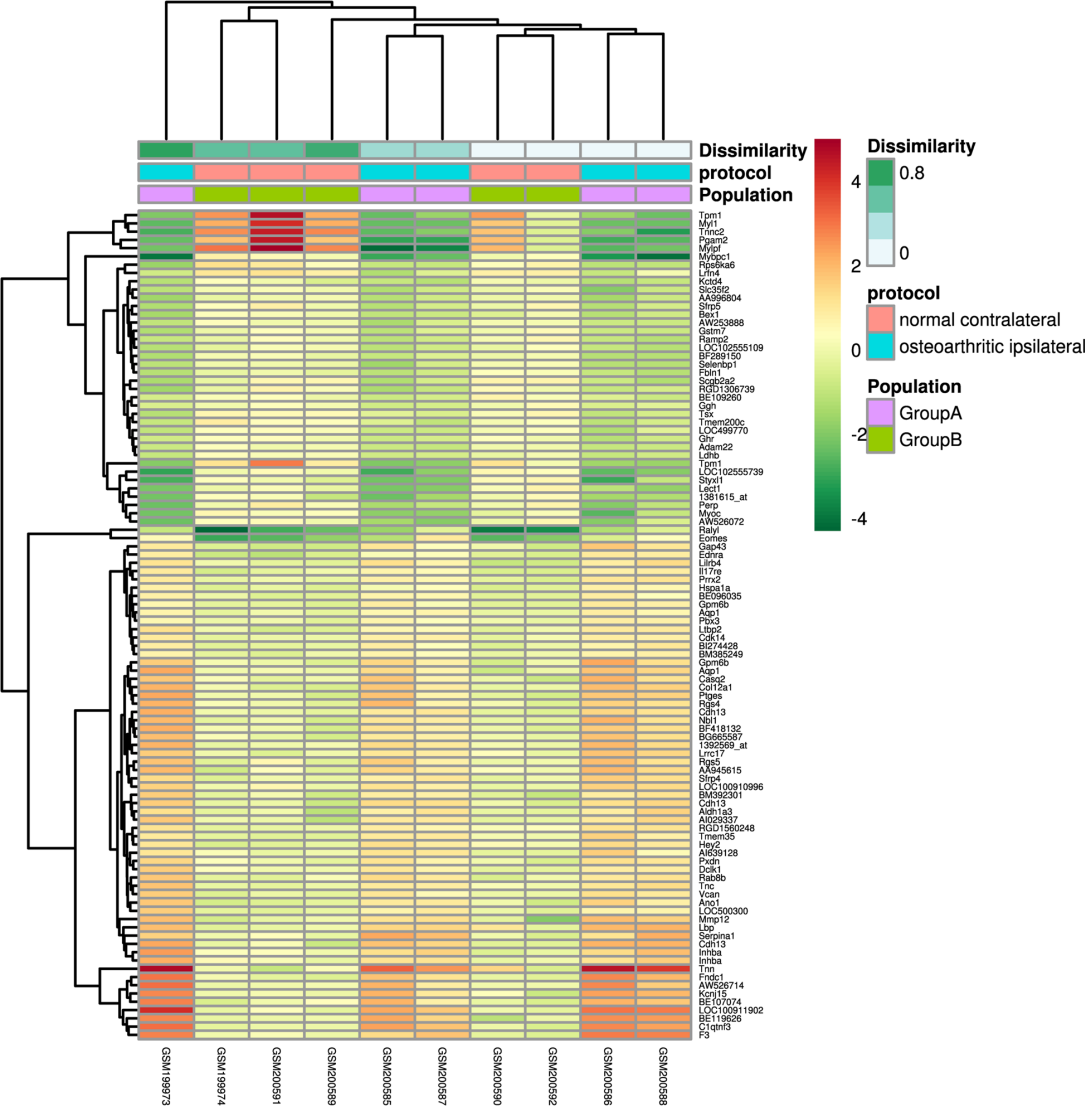
Heatmap of DEGs in comparison between OA group and contralateral group. The heatmap was produced using GeoDiver (https://www.geodiver.co.uk/)

Several DEGs (188 in comparison between OA and sham-operated group, and 160 in comparison between OA and contralateral group) were identified using GeoDiver according to the pre-defined criterion. The results of heatmap were showed in Fig. 1 (OA vs. sham group) and Fig. 2 (OA vs. contralateral group). Detailed DEGs of both comparison cohorts were showed in Additional file 1: Table S1.

### Enrichment analysis of DEGs

**Fig. 3:**
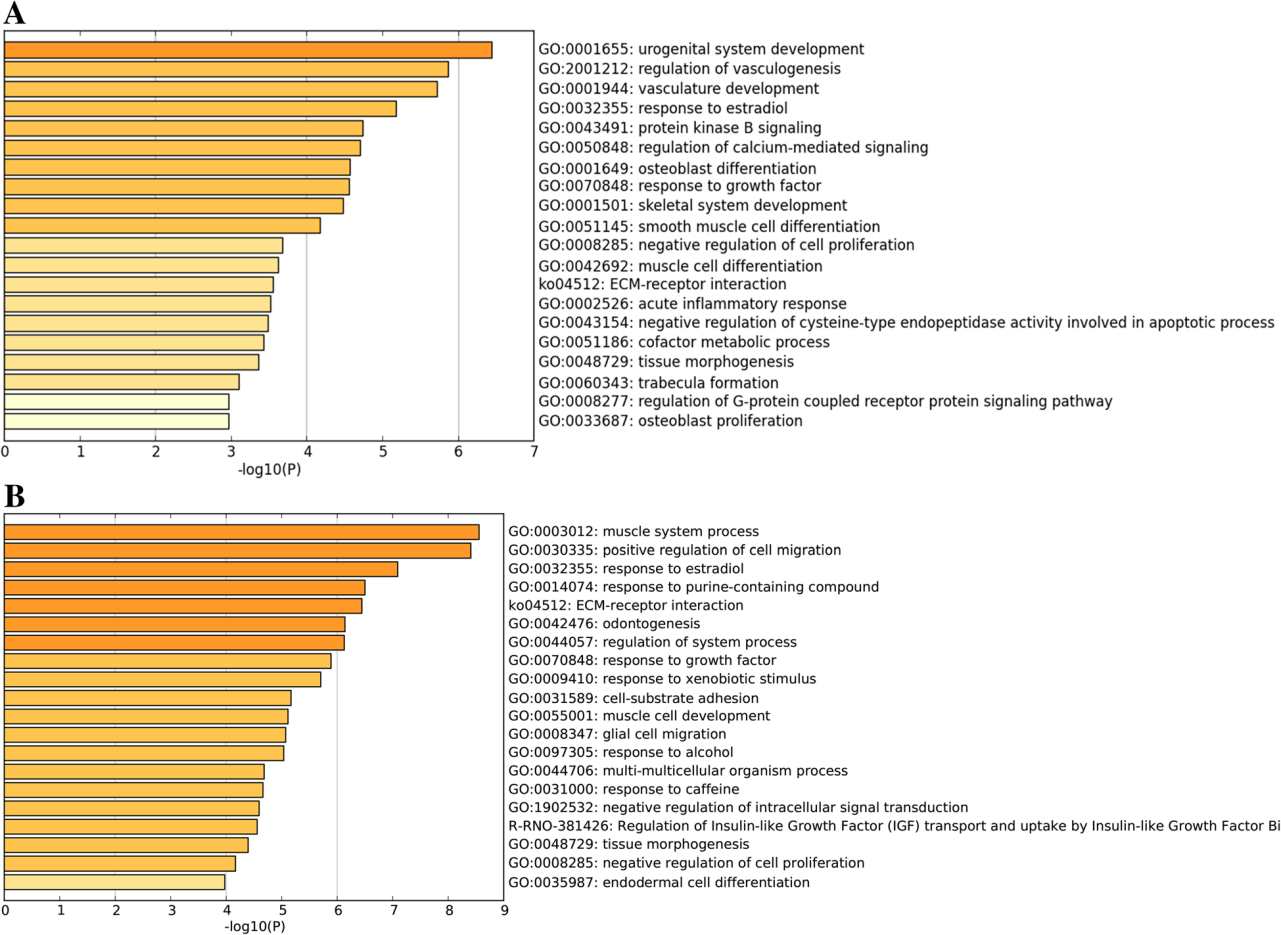
Function enrichment analysis of DEGs in comparison between OA group and control group. **a** Heatmap of top 20 enriched terms across DEGs in comparison between OA group and sham group, colored by *p* values. **b** Heatmap of top 20 enriched terms across DEGs in comparison between OA group and contralateral group, colored by *p* values. The heatmaps (**a**, **b**) were produced using Metascape (http://metascape.org/gp/index.html#/main/step1)

**Fig. 4:**
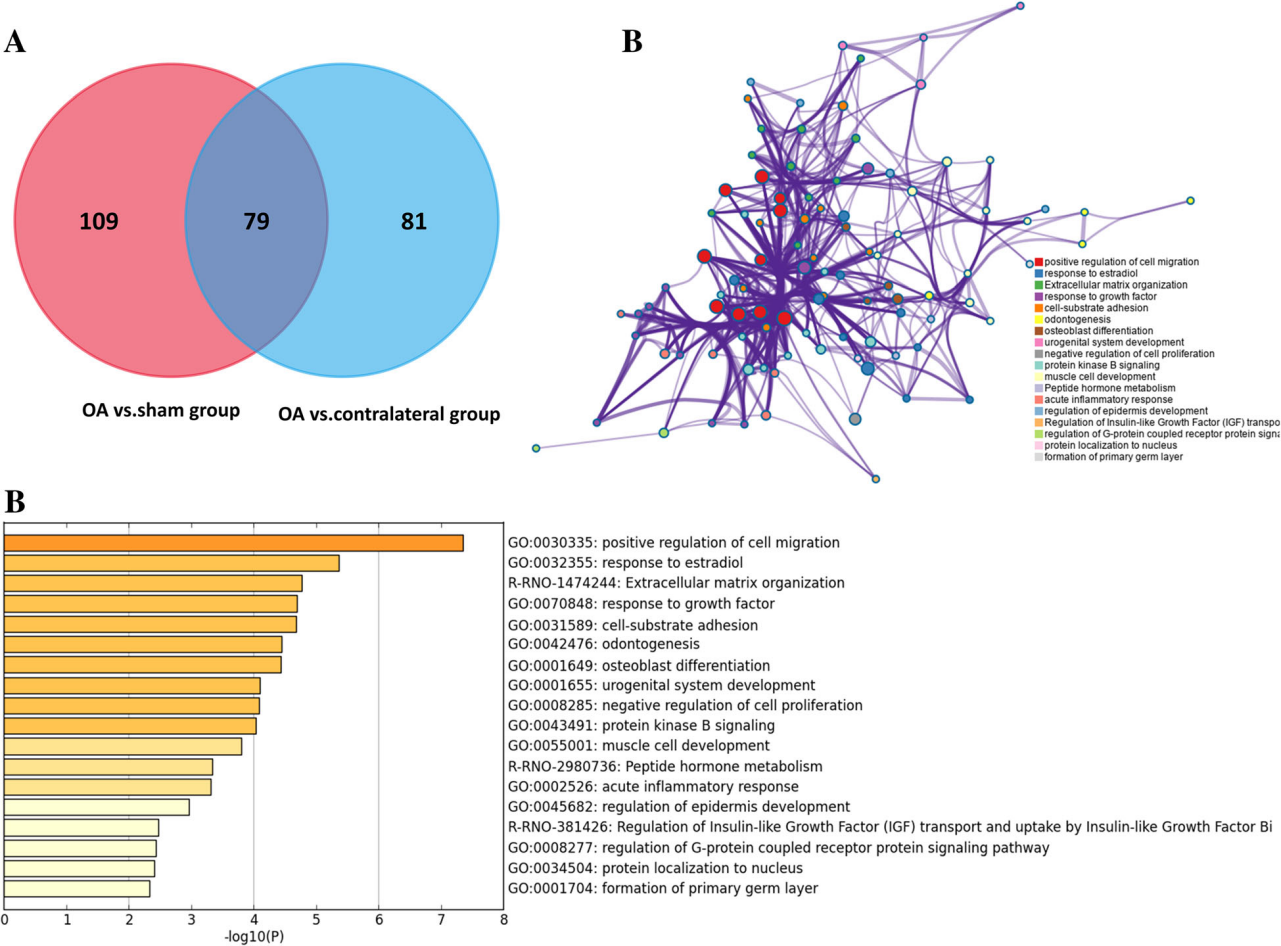
Function enrichment analysis of common DEGs in comparison between OA group and control groups. **a** Venn diagram revealed that 79 common DEGs were differently expressed in two comparison cohorts. **b** Heatmap of enriched terms across common DEGs in comparison between OA group and control groups, colored by *p* values. **c** Network of enriched terms are colored by cluster ID and nodes share the same cluster are typically close to each other. The Venn graph (**a**) was produced using Funrich (http://www.funrich.org/). **b**, **c** Produced using Metascape (http://metascape.org/gp/index.html#/main/step1)

**Fig. 5:**
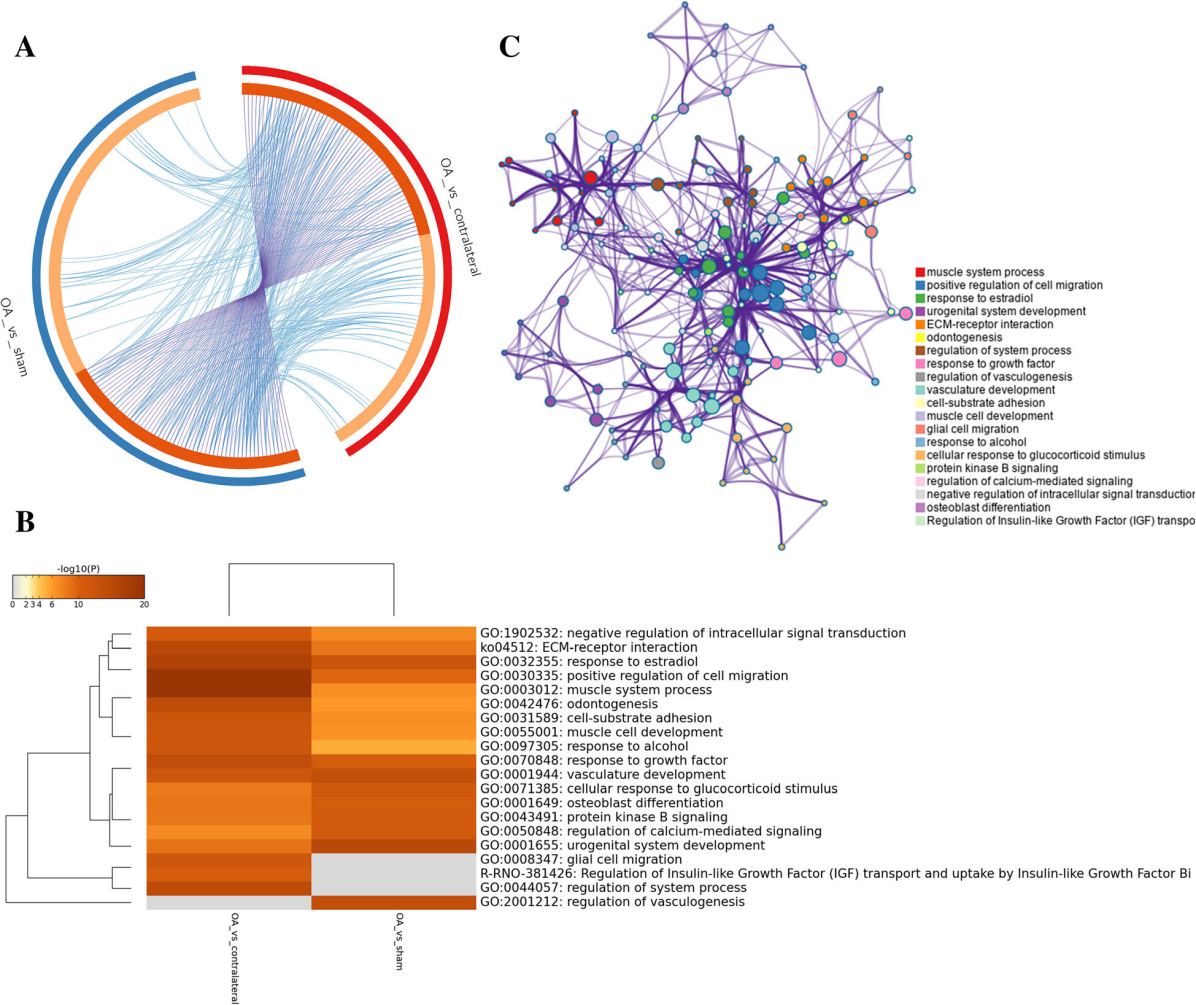
Meta-enrichment analysis summary for DEGs lists in two comparison cohorts. **a** Overlap among gene lists at the gene level, where purple curves link identical genes and blue curves link genes belong to the same enriched ontology term. The inner circle represents gene lists, where hits are arranged along the arc. Genes hit multiple lists are colored in dark orange, and genes unique to a list are shown in light orange. **b** Heatmap of top 20 enriched terms across two gene lists, colored by *p* values. **c** Network of enriched terms are colored by cluster ID and nodes share the same cluster are typically close to each other. **a**–**c** Produced using Metascape (http://metascape.org/gp/index.html#/main/step1)

DEGs in comparison between OA and sham group mainly enriched in urogenital system development, regulation of vasculogenesis, vasculature development, response to growth factor, negative regulation of cell proliferation, skeletal system development (GO), and ECM-receptor interaction (KEGG) (Fig. 3a). Furthermore, DEGs in comparison between OA and contralateral group mainly enriched in muscle system process, positive regulation of cell migration, regulation of system process, response to growth factor (GO), and ECM-receptor interaction (KEGG) (Fig. 3b). Two comparison cohorts shared 79 intersection genes, which mainly enriched in positive regulation of cell migration, response to growth factor, negative regulation of cell proliferation, response to estradiol, cell-substrate adhesion (GO), and Extracellular matrix organization (Reactome Gene Sets) (Fig. 4a–c). Additionally, we also performed meta-enrichment analysis based on two DEGs lists, which suggested that DEGs from two comparison cohorts mainly enriched in vasculature development, positive regulation of cell migration, response to growth factor (GO), and ECM-receptor interaction (KEGG) (Fig. 5a–c).

### PPI network analysis of DEGs

**Fig. 6:**
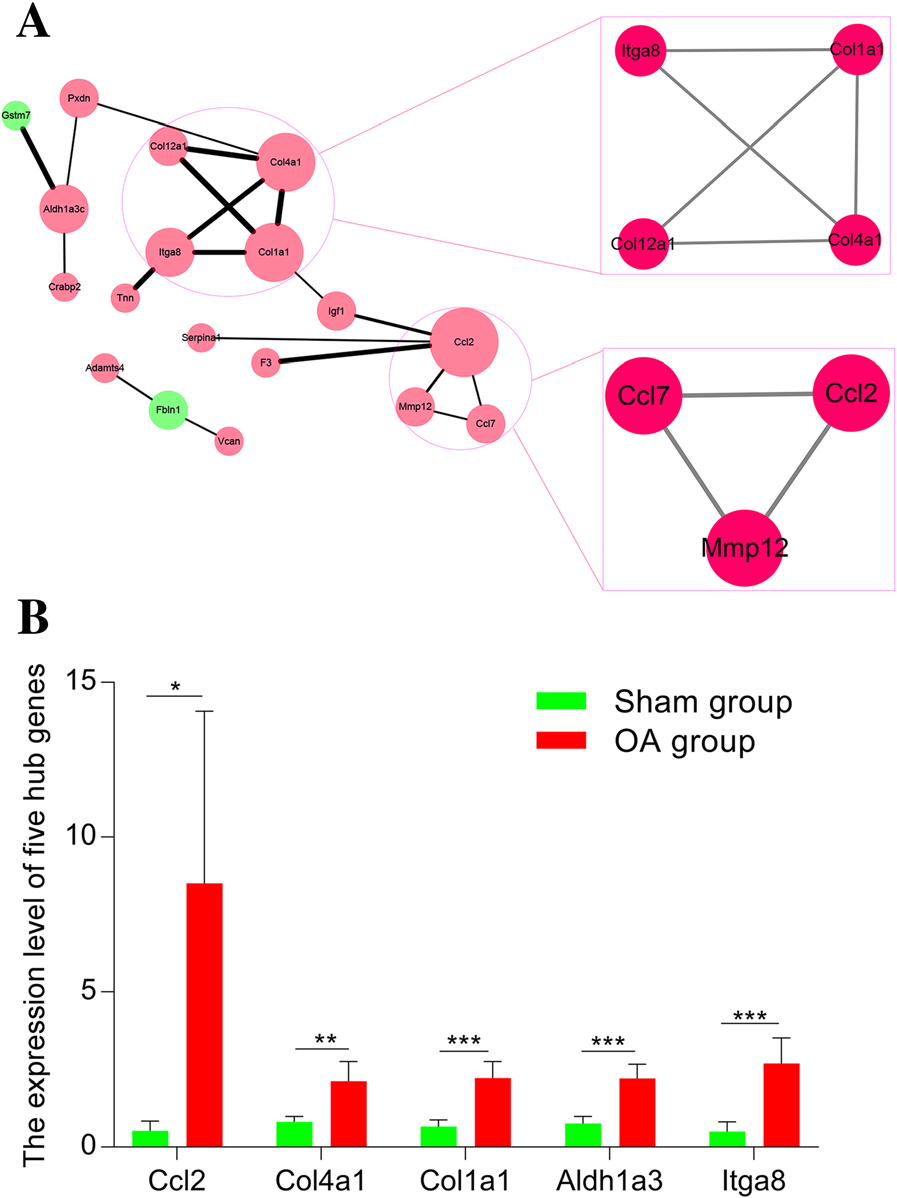
PPI network analysis and hub genes in the protein–protein interaction network. **a** The protein–protein interaction network and sub-modules for DEGs. Red circle denotes upregulated genes; green circle denotes downregulated genes; the size of circle denotes centrality degree of DEGs; the width of edge denotes combined score of gene-gene interaction. **b** The expression level of five hub genes in OA group and sham group. The original expression values of five hub genes are extracted from GEO Profiles (https://www.ncbi.nlm.nih.gov/geoprofiles/)

PPI network analysis of intersection genes contained 18 nodes and 19 edges (Fig. 6a). Centiscape 2.2 was employed to analyze the PPI network and five genes (Ccl2, Col4a1, Col1a1, Aldh1a3, and Itga8) with centrality degree ≥ 3 were defined as key genes (Table 1). Also, MCODE plugin was used to extract sub-network modules. Two sub-networks were screened out and contained 7 DEGs (Itga8, Col1a1, Col12a1, Col4a1, Ccl7, Mmp12, and Ccl2) (Fig. 6a). The expression levels of five hub genes in OA group were higher than those in sham group (Fig. 6b) (Additional file 2: Table S2).

**Table 1:** The statistical results of the connectivity degrees of the PPI network

Gene	Degree	logFC (OA vs. Sham group)	adj.P.Val (OA vs. Sham group)
Ccl2	5	3.907	0.014
Col4a1	4	1.331	0.026
Col1a1	4	1.759	0.014
Aldh1a3	3	1.53	0.015
Itga8	3	2.598	0.019
Col12a1	2	1.703	0.009
Igf1	2	2.508	0.006
Mmp12	2	1.879	0.026
Fbln1	2	− 1.376	0.006
Ccl7	2	3.02	0.015
Pxdn	2	1.266	0.026
Gstm7	1	− 1.076	0.023
Tnn	1	4.65	0.005
F3	1	2.545	0.009
Adamts4	1	2.042	0.006
Crabp2	1	3.089	0.011
Serpina1	1	1.294	0.017
Vcan	1	1.13	0.023

### MiRNA-target regulatory network

**Fig. 7:**
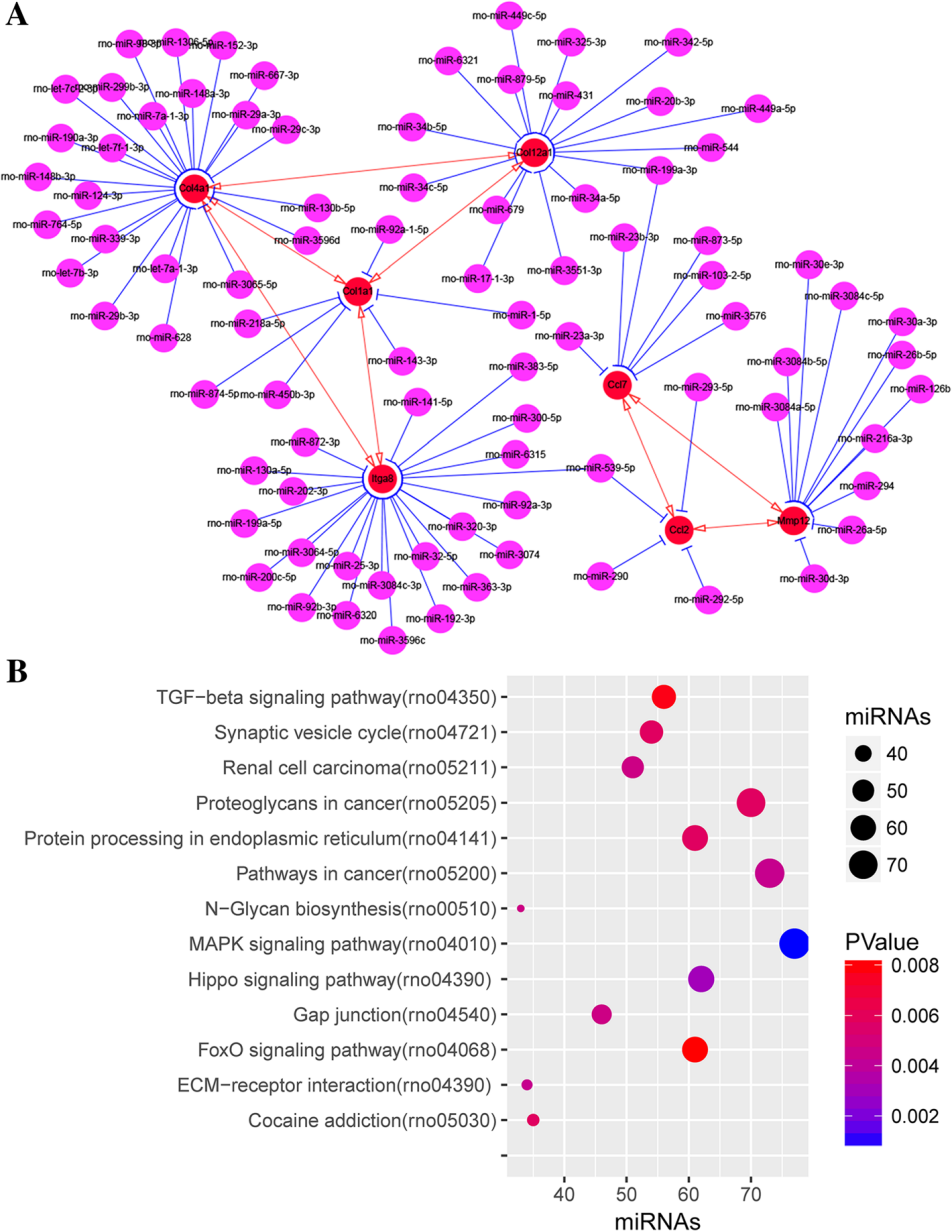
MiRNA–mRNA regulatory networks in surgical-induced rat OA model. **a** MiRNA–mRNA (Itga8, Col1a1, Col12a1, Col4a1, Ccl7, Mmp12, and Ccl2) regulatory networks. Red nodes denote genes from sub-networks and purple nodes denote miRNAs; red edges denote mRNAs-mRNAs interaction relationship and blue edges denote miRNAs-mRNAs interaction relationship. **b** Bubble graph for KEGG pathway enrichment analysis of predicted miRNAs

MiRNAs binding to DEGs in sub-networks were predicted using miRDB. The miRNA-mRNA regulatory network included 106 nodes and 96 edges. Of these, rno-miR-539-5p can antagonize both Ccl2 and Itga8 and rno-miR-199a-3p can antagonize both Ccl7 and Col12a1 (Fig. 7a). Also, we performed functional enrichment analysis of these predicted miRNAs, which mainly enriched in MAPK signaling pathway, pathways in cancer, proteoglycans in cancer, Hippo signaling pathway, and FoxO signaling pathway (KEGG) (Fig. 7b).

## Discussion

The surgical-induced OA rat model is one of the most common animal models in vivo. Therefore, it is essential to unveil potential molecular mechanisms in a surgical-induced OA rat model, which will contribute to clarifying the pathogenesis of OA. In the current study, several key genes and pathways were identified through reanalyzing GSE8077 dataset using integrated bioinformatics analysis.

Three steps were followed to perform enrichment analysis of DEGs, which revealed that DEGs mainly enriched in vasculature development, response to growth factor, positive regulation of cell migration, and ECM-receptor interaction. Many studies found that vasculature development at the osteochondral junction and synovium was associated with the onset and development of OA [29, 30]. Meanwhile, some growth factors, such as VEGF and NGF, were obviously upregulated in subchondral spaces, vascular channels and chondrocytes of OA patients [31]. Vasculature development was usually activated by some growth factors, such as VEGF [32]. Further studies also indicated that repression for angiogenesis in osteochondral junction and synovium may had a potential inhibitory influence on OA progression [33]. Accordingly, these growth factors may act as potential therapeutic targets for OA. Generally, loss of cartilage homeostasis and the dysfunction of chondrocytes phenotypes including cell apoptosis, cell migration, and cell proliferation are the critical pathological process of OA [34, 35]. The current study also revealed that DEGs enriched in positive regulation of cell migration and negative regulation of cell proliferation. Meanwhile, ECM-receptor interaction was found to be the most significantly enriched pathway for DEGs, which was further verified by many previous studies [36, 37]. Therefore, dysfunction of these cell phenotypes and molecules may play important roles in OA development and can act as promising pathological signatures for OA in vitro and in vivo.

Many OA-related key genes were also identified in the surgical-induced rat model, including Ccl2, Col4a1, Col1a1, Aldh1a3, and Itga8. Integrin α8 (Itga8) was an important component of ECM-receptor interaction pathway. It was significantly upregulated in mesenchymal cells and played important roles in the expression of extracellular matrix components [38]. Gong et al. revealed that Itga8 may participate in the degradation of extracellular matrix, including collagen type XI alpha 1, aggrecan, collagen type VI alpha 1 in periodontal ligament tissues [39]. Considering that imbalance of extracellular matrix anabolism and catabolism was the critical pathological process in OA, it was worthwhile to explore the potential roles of Itga8 in OA. Several studies revealed that the abnormal expression of collagen-related genes (Col1a1, Col4a1 and MMP12) participated in the pathogenesis of OA onset and progression, which were consistent with our study [40–43]. Our study also found that CCL2 was upregulated in surgical-induced rat OA model and may participate in OA pathogenesis, which was further supported by previous studies [44, 45]. Recent studies revealed that CCL2 can be responsible for monocytes’ migration and cartilage degeneration, and the CCL2/CCR2 axis may play a critical role in OA-related pain [46, 47]. As one of Aldehyde dehydrogenase isoforms, Aldh1a3 was obviously upregulated in human articular chondrocytes. Furthermore, the activation of Aldh1a3 may be responsible for the producing activity of collagen II in chondrocytes [48]. Thus, whether the dysfunction of Aldh1a3 was associated with OA pathogenesis was worthwhile to be further explored. Collectively, considering that the important roles of these key genes in OA, they may be used as potential molecular biomarkers and therapeutic targets for OA.

Previous studies revealed that many differentially expressed miRNAs (for example, miR-145 and miR-140) were associated with OA development and progression [49, 50]. Also, some researchers demonstrated that circulating miRNAs, such as miR-19b-3p, miR-122-5p, miR-486-5p, hsa-miR-140-3p, hsa-miR-671-3p, and hsa-miR-33b-3p, can be promising diagnostic biomarkers for knee OA [36, 51]. In the current study, we identified that 99 predicted miRNAs were mainly enriched in MAPK signaling pathway and of these, miR-199a-3p and miR-539-59 may act as potential key miRNAs to regulate corresponding mRNAs. Actually, previous studies have verified that these predicted miRNAs and pathways played important roles in OA. For instance, Sun et.al. found that inhibition of P38-MAPK signaling pathway participated in repressing chondrocytes apoptosis and the release of proinflammatory cytokines in OA [52]. Furthermore, Akhtar et.al. suggested that overexpression of miR-199a can inhibit MAPK signaling pathway, thus attenuating OA progression [53]. Therefore, these important miRNAs and signaling pathways can be served as potential diagnostic biomarkers and therapeutic targets for OA, which may provide potential hallmarks for further experimental studies.

The strength of the current study was that we performed comprehensive enrichment analysis based on Metascape in a rat OA model. Apart from common DEGs between OA group and control groups, we also performed meta-enrichment analysis of all the DEGs in two comparison cohorts. Besides, pathway enrichment analyses were undertaken to explore the potential roles of predicted miRNAs. Regardless of aforementioned strengths, our studies also existed some limitations. Firstly, our findings were merely based on limited sample size (five in each group), so it was hard to exclude potential random error and false positive. Accordingly, further studies with large sample size should be warranted. Secondly, the results of the current study were totally based on bioinformatics prediction and lacked subsequent experimental verification, such as RT-qPCR, western blot, and immunohistochemistry. Actually, owing to limited available materials in the current study, it was hard for us to verify our findings with these experiments. Anyway, the current study may provide some potential useful orientation for future experimental studies*.*

## Conclusions

The current study shows that some key genes and pathways, such as Ccl2, Col4a1, Col1a1, Aldh1a3, Itga8, ECM-receptor interaction, and MAPK signaling pathway may be associated with OA progression and act as potential biomarkers and therapeutic targets for OA. These findings need further experimental verification, but may provide potential useful evidence for future researches in OA.

## Additional files

Additional file 1: **Table S1.** Detailed DEGs in a surgical-induced rat OA model. (XLSX 12 kb)

Additional file 2: **Table S2.** The original expression value of five hub genes extracted from GEO Profiles. (DOCX 19 kb)

## Abbreviations

DEGs: Differentially expressed genes
GO: Gene ontology
KEGG: Kyoto Encyclopedia of Genes and Genomes
MCODE: Molecular complex detection
PPI: Protein–protein interaction
RT-qPCR: Real-time quantitative PCR
