# Supplementary material for: Transcriptome analyses identify key genes and potential mechanisms in a rat model of osteoarthritis
Source: J Orthop Surg Res. 2018 Dec 14;13:319. doi: 10.1186/s13018-018-1019-3 (PMC6295024; doi:10.1186/s13018-018-1019-3)
Supplement: Supplementary file 2 — Table S2. The original expression value of five hub genes extracted from GEO Profiles. (DOCX 19 kb) [file 13018_2018_1019_MOESM2_ESM.docx]

| **Col1a1*** |  |  |  |
| --- | --- | --- | --- |
| Sample | Title | Value | Rank |
| GSM200584 | Sham 1 | 0.42 | 11 |
| GSM200593 | Sham 2 | 0.572 | 15 |
| GSM200594 | Sham 3 | 0.603 | 15 |
| GSM200595 | Sham 4 | 1 | 46 |
| GSM200596 | Sham 5 | 0.729 | 21 |
| GSM199974 | Contralateral 1 | 0.853 | 34 |
| GSM200589 | Contralateral 2 | 1.38 | 92 |
| GSM200590 | Contralateral 3 | 0.948 | 45 |
| GSM200591 | Contralateral 4 | 0.479 | 12 |
| GSM200592 | Contralateral 5 | 1.021 | 62 |
| GSM199973 | OA 1 | 2.641 | 99 |
| GSM200585 | OA 2 | 1.656 | 97 |
| GSM200586 | OA 3 | 2.796 | 99 |
| GSM200587 | OA 4 | 1.658 | 98 |
| GSM200588 | OA 5 | 2.316 | 99 |
| *https://www.ncbi.nlm.nih.gov/geo/tools/profileGraph.cgi?ID=GDS2809:1388116_at | | | |
|  |  |  |  |
| **Ccl2*** |  |  |  |
| Sample | Title | Value | Rank |
| GSM200584 | Sham 1 | 0.444 | 12 |
| GSM200593 | Sham 2 | 0.633 | 17 |
| GSM200594 | Sham 3 | 0.398 | 10 |
| GSM200595 | Sham 4 | 1 | 46 |
| GSM200596 | Sham 5 | 0.186 | 4 |
| GSM199974 | Contralateral 1 | 0.972 | 48 |
| GSM200589 | Contralateral 2 | 0.337 | 8 |
| GSM200590 | Contralateral 3 | 1.055 | 70 |
| GSM200591 | Contralateral 4 | 0.773 | 24 |
| GSM200592 | Contralateral 5 | 1.528 | 96 |
| GSM199973 | OA 1 | 16.28 | 100 |
| GSM200585 | OA 2 | 3.388 | 99 |
| GSM200586 | OA 3 | 10.54 | 99 |
| GSM200587 | OA 4 | 2.861 | 99 |
| GSM200588 | OA 5 | 9.499 | 99 |
| *https://www.ncbi.nlm.nih.gov/geo/tools/profileGraph.cgi?ID=GDS2809:1367973_at | | | |
|  |  |  |  |
| **Col4a1*** |  |  |  |
| Sample | Title | Value | Rank |
| GSM200584 | Sham 1 | 0.811 | 29 |
| GSM200593 | Sham 2 | 0.661 | 18 |
| GSM200594 | Sham 3 | 0.656 | 17 |
| GSM200595 | Sham 4 | 0.995 | 45 |
| GSM200596 | Sham 5 | 1 | 55 |
| GSM199974 | Contralateral 1 | 1.017 | 59 |
| GSM200589 | Contralateral 2 | 0.763 | 26 |
| GSM200590 | Contralateral 3 | 0.73 | 21 |
| GSM200591 | Contralateral 4 | 1.033 | 57 |
| GSM200592 | Contralateral 5 | 0.969 | 46 |
| GSM199973 | OA 1 | 3.017 | 99 |
| GSM200585 | OA 2 | 1.701 | 98 |
| GSM200586 | OA 3 | 2.399 | 99 |
| GSM200587 | OA 4 | 1.37 | 93 |
| GSM200588 | OA 5 | 2.092 | 99 |
| *https://www.ncbi.nlm.nih.gov/geo/tools/profileGraph.cgi?ID=GDS2809:1373245_at | | | |
|  |  |  |  |
| **Aldh1a3*** |  |  |  |
| Sample | Title | Value | Rank |
| GSM200584 | Sham 1 | 0.529 | 15 |
| GSM200593 | Sham 2 | 0.744 | 23 |
| GSM200594 | Sham 3 | 1 | 55 |
| GSM200595 | Sham 4 | 1.005 | 51 |
| GSM200596 | Sham 5 | 0.595 | 15 |
| GSM199974 | Contralateral 1 | 0.858 | 34 |
| GSM200589 | Contralateral 2 | 0.484 | 12 |
| GSM200590 | Contralateral 3 | 0.868 | 33 |
| GSM200591 | Contralateral 4 | 1.022 | 56 |
| GSM200592 | Contralateral 5 | 0.822 | 27 |
| GSM199973 | OA 1 | 2.354 | 98 |
| GSM200585 | OA 2 | 1.743 | 98 |
| GSM200586 | OA 3 | 1.788 | 97 |
| GSM200587 | OA 4 | 2.224 | 99 |
| GSM200588 | OA 5 | 2.9 | 99 |
| * https://www.ncbi.nlm.nih.gov/geo/tools/profileGraph.cgi?ID=GDS2809:1383469_at | | | |
|  |  |  |  |
| **Itga8*** |  |  |  |
| Sample | Title | Value | Rank |
| GSM200584 | Sham 1 | 0.311 | 8 |
| GSM200593 | Sham 2 | 0.199 | 4 |
| GSM200594 | Sham 3 | 0.378 | 9 |
| GSM200595 | Sham 4 | 0.615 | 16 |
| GSM200596 | Sham 5 | 1 | 55 |
| GSM199974 | Contralateral 1 | 1.25 | 83 |
| GSM200589 | Contralateral 2 | 1.185 | 83 |
| GSM200590 | Contralateral 3 | 0.814 | 27 |
| GSM200591 | Contralateral 4 | 0.787 | 25 |
| GSM200592 | Contralateral 5 | 0.505 | 12 |
| GSM199973 | OA 1 | 2.455 | 98 |
| GSM200585 | OA 2 | 3.167 | 99 |
| GSM200586 | OA 3 | 3.821 | 99 |
| GSM200587 | OA 4 | 1.655 | 98 |
| GSM200588 | OA 5 | 2.379 | 99 |
| [*https://www.ncbi.nlm.nih.gov/geo/tools/profileGraph.cgi?ID=GDS2809:1383398_at](https://www.ncbi.nlm.nih.gov/geo/tools/profileGraph.cgi?ID=GDS2809:1383398_at) | | | |
|  |  |  |  |
